# Donald J. Krogstad, MD (1943–2020), Physician-Scientist, Malaria Researcher, and Mentor

**DOI:** 10.4269/ajtmh.20-1943

**Published:** 2020-09-10

**Authors:** Ousmane Koita, Seydou Doumbia, Richard Oberhelman, Peter Weller, Joseph Keating, Thomas Eisele

**Affiliations:** 1 Laboratory of Applied Molecular Biology, Faculty of Pharmacy, University of Sciences, Techniques and Technologies of Bamako, Bamako, Mali;; 2 Faculty of Medicine and Odontostomatology, University of Sciences, Techniques and Technology of Bamako, Bamako, Mali;; 3 Department of Tropical Medicine, Tulane School of Public Health and Tropical Medicine, New Orleans, Louisiana;; 4 Harvard Medical School, Harvard T.H. Chan School of Public Health, Boston, Massachusetts

We are deeply saddened to announce the death of Donald Krogstad, MD, FASTMH, past-president of the American Society of Tropical Medicine and Hygiene (ASTMH), on August 14, 2020 in Palm Coast, FL. He is survived by his wife Frances Krogstad and by two sons, Aric and Kirk. Fran Krogstad was a steadfast partner in their professional and personal lives, who gave of herself to facilitate his work and support his many collaborators and trainees in the United States, West Africa, and around the world.

Don Krogstad pursued a career as a physician-scientist and tropical infectious disease researcher starting in 1969 with a 2-month Harvard Medical School elective at the Hôpital Albert Schweitzer in Deschapelles, Haiti. He completed an internship and residency in internal medicine (1969–1971; 1975–1976) as well as a fellowship in infectious diseases (1976–1978) at the Massachusetts General Hospital, Boston, MA. While on leave from his residency, he completed 2 years in the Epidemic Intelligence Service at the CDC (1971–1973) and volunteer physician service through the U.S. Peace Corps in Lilongwe, Malawi (1973–1975). He joined the faculty of the Washington University School of Medicine in 1978 and moved to the Department of Tropical Medicine at the Tulane School of Public Health and Tropical Medicine in 1992, where he served as chair for over 15 years.

Don had long-lasting contributions to the ASTMH. He served as Scientific Program Chair from 1984 to 1989. He was a councilor from 1989 to 1990 and served as the ASTMH president in 1992. Acting as more than the customary titular president, he had the novel idea of convening a “retreat” that would lead the ASTMH to consider relevant issues and its future roles. Out of this retreat was an ASTMH embracement of encouraging and developing new pathways/programs focused on enhancing training programs in clinical tropical medicine. These coordinated programs to advance clinical education in tropical medicine, as then envisioned and embraced, continue to this day in ASTMH activities, as an important part of Don’s legacy.

Don is best known for his contributions to research on the pathogenesis, epidemiology, and treatment of malaria, founded on a long-standing partnership with public health leaders from Mali that originated in the early 1990s with the NIH-funded Malaria Research and Training Center at the University of Bamako. More than a dozen Malian scientists have been trained at Tulane University through this collaboration to address the need for academic leadership in Africa. His numerous former trainees remember his lifelong commitment to training and mentoring African scientists in the fight against malaria. His collaborations with Malian scientists led to the establishment of many successful malaria control programs that built research capacity and infrastructure in Mali, including the NIH-funded Tropical Medicine Research Center for malaria (1996–2001), the Laboratory of Molecular Biology, the West African Center of Excellence in Malaria Research (2010–2016), the Mali International Center of Excellence in Malaria Research awarded in 2017, and the National Institute of Allergy and Infectious Diseases Tropical Medicine Research Center on Neglected Tropical Diseases (2012–2017).

Don also collaborated with Dr. Ousmane Koita to create the first clinical trial unit in Mali at the University Clinical Research Center at Point G Hospital, where he conducted a phase 2 trial of the candidate antimalarial AQ-13, developed by his group at Tulane, under the Investigational New Drug Program at the U.S. Food and Drug Administration. Working since the mid-1990s to take this compound from a laboratory in the United States to the field in Mali, he demonstrated that AQ-13 is as efficacious as artemether + lumefantrine to treat uncomplicated malaria. In doing so, he created a new paradigm for malaria research in Mali by linking the laboratory with the field, facilitated by technology transfer that revolutionized information technology and laboratory capacity, resulting in new programs for molecular diagnosis of malaria, geographic information system disease mapping, bioinformatics research training, and research on informed consent in illiterate populations.

Don’s former students and trainees remember him as a brilliant scientist who coached and mentored aspiring trainees from all over the world on how to harmonize the laboratory with malaria field research. As a visionary, he had a keen ability to foster and develop an individual’s capacity for public health research, always with the aim of enabling young scientists to stand on their own feet. Don built a bridge between the tropics in West Africa and New Orleans, allowing many African public health leaders to be trained at Tulane and many Americans to experience field research in Mali. He continued mentoring young scientists around the world until his death. For all of us, Don brought the light that is paving our ways. We will work to achieve his goal of a world without malaria, with a focus on training African scientists in infectious diseases. We will always remember him with his notepad and pen, always listening and gaining knowledge from everyone. You will be missed, Dr. Krogstad.

**Figure:**
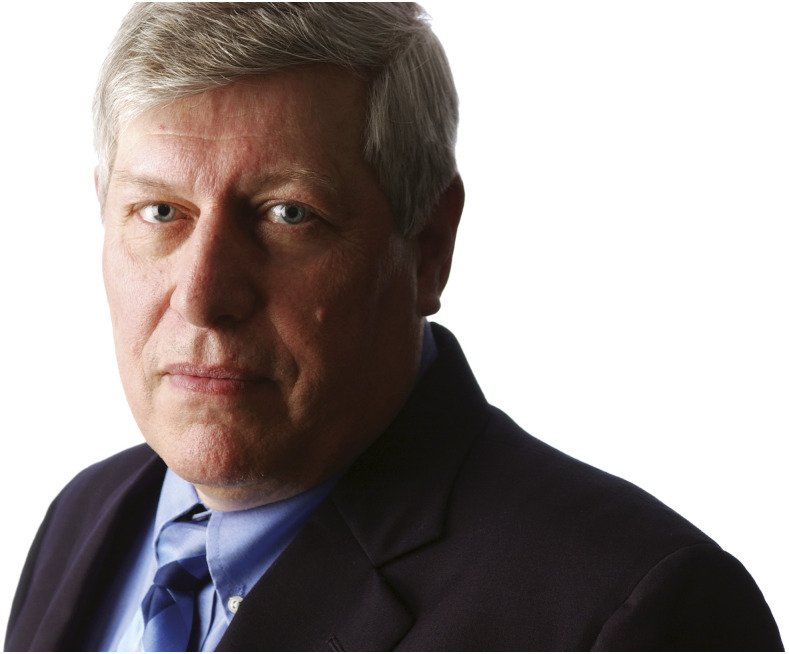
Donald J. Krogstad, MD, FASTMH

